# Tropisms of the Dowser Texture

**DOI:** 10.3390/ma13204681

**Published:** 2020-10-21

**Authors:** Pawel Pieranski, Maria Helena Godinho

**Affiliations:** 1 Laboratoire de Physique des Solides, Université Paris-Saclay, Bât. 510, 91405 Orsay, France; 2 Departamento Ciência dos Materiais, CENIMAT, Faculdade de Ciências e Tecnologia, Universidade Nova de Lisboa, 2829-516 Caparica, Portugal; mhg@fct.unl.pt

**Keywords:** nematic liquid crystal, dowser texture, bowser texture, complex order parameter, XY order parameter, topological defects, monopole, flexo-electricity

## Abstract

Due to its low symmetry $C_{2v}$, the dowser texture is characterised by a 2D unitary vector field or alternatively by a unitary complex field. For the same symmetry reasons, the dowser texture is sensitive, in first order, to perturbations such as thickness gradients, electric fields or flows. We will focus on corresponding properties called respectively: cuneitropism, electrotropism and rheotropism. In particular we will show that topological defects, known as dowsons or monopoles, can be manipulated by means of these tropisms.

## 1. Sensitivity of Systems Resulting from Symmetry Breakings

**Figure 1:**
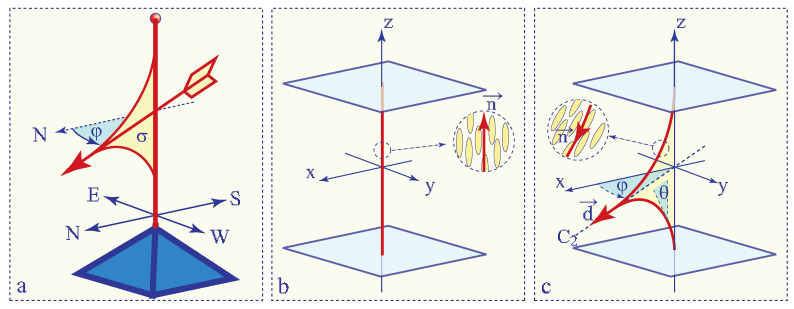
Sensitivity of systems with degenerated order parameters. (**a**) The azimuthal orientation of a weather vane is degenerated so that it is responsive to aerodynamic torques. (**b**) Homeotropic texture. In the absence of surfaces and fields, orientation of the director field **n** would be arbitrary. Therefore, surfaces treated for homeotropic anchoring impose easily the uniform orientation **n**//**z**. (**c**) Director field **n**, symmetry C${}_{2v}$ and order parameter **d** (or φ) of the dowser texture. The group C${}_{2v}$ contains three symmetry elements: the twofold axis C${}_{2}$, the mirror plane σ and the mirror plane (x,y). By analogy with the weather vane, the dowser texture is sensitive in first order to fields such as a Poiseuille flow.

The weather vane shown in Figure 1a is sensitive to winds because its shape has a very low symmetry (mirror plane σ) and its azimuthal orientation is degenerated so that it can be easily imposed by aerodynamic torques.

Similarly, certain systems resulting from symmetry breaking are naturally responsive to stimuli because their order parameters are degenerated. For example, in uniaxial nematics, the quadrupolar order parameter [1]

(1) $$Q_{\alpha \beta }=Q\left(n_{\alpha }n_{\beta }-\frac{1}{3}\delta_{\alpha \beta }\right)$$

resulting from the $O\left(3\right)\Rightarrow D_{\infty h}$ symmetry breaking at the Isotropic ⇒ Nematic phase transition is degenerated with respect to all possible orientations of the uniaxial symmetric $Q_{\alpha \beta }$ tensor collinear with the director **n**. For this reason, the otherwise arbitrary orientation of the director **n** can be imposed without any energy expense by action of surfaces or fields.

In particular, it is well known that surfaces of glass slides coated with e.g., an egg-yolk lecithin orient molecules of the nematic in contact with them in the orthogonal direction z. Usually, due to the action of such a so-called homeotropic anchoring, the director field in bulk will adopt the same direction **n**//**z** (see Figure 1b).

In the absence of surfaces, electric or magnetic fields can also orient the director **n**. Due to the $D_{\infty h}$ symmetry of the nematic order parameter $Q_{\alpha \beta }$, its lowest order coupling with the electric and magnetic fields is expressed by torques [1]:

(2) $$\Gamma_{a}=\epsilon_{o}\epsilon_{a}\left(n\cdot E\right)\left(n\times E\right)\;\mathrm{and}\;\Gamma_{a}=\frac{\chi_{a}}{\mu_{o}}\left(n\cdot B\right)\left(n\times B\right)$$

involving the dielectric and magnetic anisotropies $\epsilon_{a}=\epsilon_{//}-\epsilon_{⊥}$ and $\chi_{a}=\chi_{//}-\chi_{⊥}$. In 5CB, these two anisotropies are positive so that the director **n** tends to be collinear with fields **E** and **B** but is not sensitive to their sign.

## 2. The Dowser and Homeotropic Textures

As already mentioned above, in a nematic confined between two parallel surfaces with homeotropic anchoring conditions, the director field takes usually a uniform direction normal to them **n**(x,y,z) = (0,0,1) (see Figure 1b). The nematic symmetry $D_{\infty h}$ is preserved in such a uniformly oriented layer which will be called in what follows **homeotropic texture**.

On the other hand, it has been known for decades that beside this ground state, another director field shown in Figure 1c and, expressed as

(3) n=[cos(πz/h)cosφ,cos(πz/h)sinφ,sin(πz/h)]

is also compatible with the homeotropic boundary conditions.

It was called “H state” by Boyd et al. [2], “splay-bend state” by Cladis et al. [3], “inversion wall” by Gilli et al. [4], “quasi-planar state” or “metastable state” by Fazio et al. [5,6]. In ref. [7] we proposed to call it “**the dowser texture**” because the director field lines in it has a shape similar to that of a Y-shaped wooden dowser’s tool. This variability of the former names of the dowser texture resulted presumably from the fact that in the works quoted above the dowser texture was not studied for itself but was considered rather as a short-lived metastable texture coexisting and competing with the homeotropic one.

**Remark on the scope of this paper**: Since the invention of the method allowing to preserve indefinitely the dowser texture [7], systematic exploration of its properties became possible and several articles reporting on them have been published in last four years [7,8,9,10,11]. Our aim here is to review these achievements. In recent works of Giomi et al. [12], Emeršič et al. [13] and Tang and Selinger [14] the intrinsic importance of the dowser texture also appeared clearly. We will refer to them in Section 6.3 and Section 11.1.

## 3. Tropisms of the Dowser Texture: First Order Sensitivities to Vector Fields

The dowser texture has the symmetry $C_{2v}$ lower than $D_{\infty h}$ of the homeotropic texture. The order parameter resulting from this $D_{\infty h}\Rightarrow C_{2v}$ symmetry breaking is a two dimensional unitary vector field **d** collinear with the $C_{\infty }$ symmetry axis of $C_{2v}$ group. Equivalently, the unitary complex field $e^{i\varphi }$ characterised by the phase φ can also be used as the order parameter of the dowser texture.

As the field **d**, called **the dowser field**, is degenerate with respect to rotations around the z axis, it should be sensitive to torques exerted on it by external vector fields **f**. Without entering into details of the interaction between the two fields **d** and **f**, one can already say that these torques can be expressed as

(4) $$\vec{\Gamma }=C_{f}d\times f$$

with the coefficient C${}_{f}$ depending on the nature of interactions of the dowser texture with fields. As such torques tend to align the dowser field **d** in directions parallel or antiparallel to the fields **f**, one can say that the dowser field **d** display tropisms to fields **f**.

**Figure 2:**
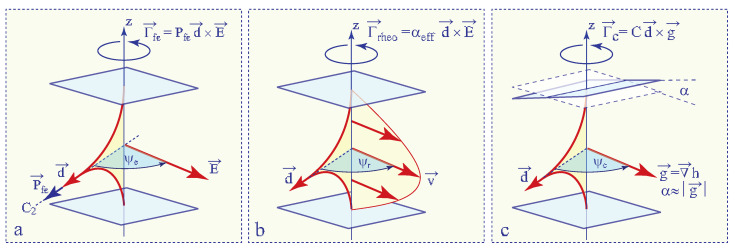
Tropisms of the dowser texture. (**a**) Electrotropism: coupling of the field **d**, via flexo-electric polarisation, with the electric field **E** [8]. (**b**) Rheotropism: hydrodynamic torque exerted on the field **d** by a Poiseuille flow [9]. (**c**) Cuneitropism: coupling of the field **d** with the thickness gradient **g** [7].

In Section 7, Section 8 and Section 9 we will focus on three tropisms represented schematically in Figure 2: (1) **Cuneitropism:** sensitivity to thickness gradients $\vec{\nabla }$h, (2) **Electrotropism**: sensitivity to the electric field **E** and (3) **Rheotropism:** sensitivity to Poiseuille flows **v**.

## 4. Sensitivity of the Dowser Texture Due to Internal Deformations

Coefficients C${}_{f}$ quantifying the tropisms of the dowser texture will be calculated in Section 7, Section 8 and Section 9 under the assumption that the polar angle θ(z)=πz/h of the director field given in Equation (3) is not affected by the action of fields. Similarly, in most of experiments discussed there the dowser texture behaves as if it had only one degree of freedom—the azimuthal angle φ.

However, in the case of the flow-assisted homeotropic⇔dowser transition discussed in Section 6.3 and Section 6.4, the pure weather vane-like response of the dowser texture to Poiseuille flows is only an approximation because simultaneously with the alignment of the field **d** in the direction of the Poiseuille flow **v**, the distribution of the polar angle θ(z) changes too upon the action of the hydrodynamic torque. For stability reason, the elastic energy $\delta F_{H/D}$ stored in the homeotropic and dowser textures must be quadratic in flow’s velocity **v**:

(5) $$\delta F_{H/D}=D_{H/D}v^{2}$$

For small flow velocities this second order term can be neglected.

## 5. Topological Defects of the Dowser Field, Nematic Monopoles

**Figure 3:**
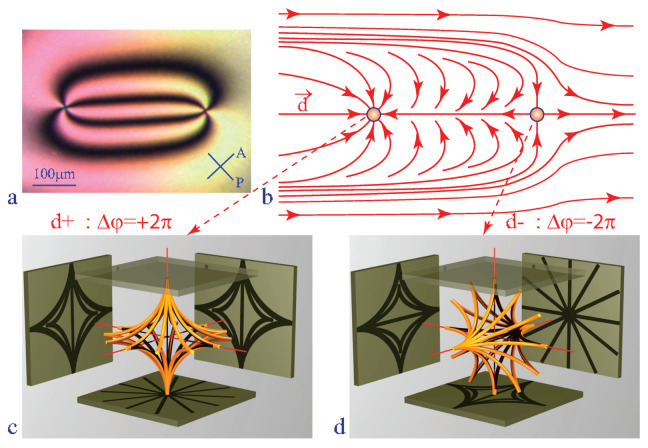
Pair of dowsons, topological defects of the dowser field **d**. (**a**) View in polarising microscope. (**b**) The dowser field **d**(x,y) inferred from (**a**). (**c**,**d**) Monopoles in the 3D director field corresponding to the dowsons d+ and d−.

Un inhomogeneous dowser field **d**(x,y) can contain point singularities called dowsons d+ and d− [11] characterised by defects of the phase Δφ=±2π on a circuit surrounding them (see Figure 3b) [10]. In three dimensions of the director field **n**(x,y,z) in the nematic layer, dowsons d+ and d− correspond to nematic monopoles [15,16,17] in the same hyperbolic configuration with different orientations shown in Figure 3c,d.

Dowsons can be generated, set into motion and brought into collisions with the help of the tropisms of the dowser texture [11].

## 6. Generation of the Dowser Texture

### 6.1. Metastability of the Dowser Texture

The dowser texture is well known to coexists with the ground state homeotropic texture immediately after fillng of a nematic cell with hoemotropic boundary conditions [18]. In these circumstances, this dowser/homeotropic coexistence appears to be almost always transitory because the distorted dowser texture is replaced by the ground state homeotropic texture. For this reason, the dowser texture was scarcely studied for itself in the past.

On the other hand it was also well known [3,4] that the dowser texture is not unstable but only metastable so that, in principle, it could be preserved indefinitely in the absence of the competing homeotropic texture. Gilli et al. [4] have shown for the first time that in thick and sufficiently small nematic droplets (called globules), hold by capillarity between glass surfaces with homeotropic anchoring conditions, the homeotropic texture collapses spontaneously into a monopole and the dowser texture (called “inversion wall” in ref. [4]) is left alone. Gilli et al. [4] have submitted such small droplets to strong enough electric fields and observed generation of additional monopoles’ pairs by electrohydrodynamic instabilities. They observed also that monopoles are set into motion by a rotating magnetic field.

**Figure 4:**
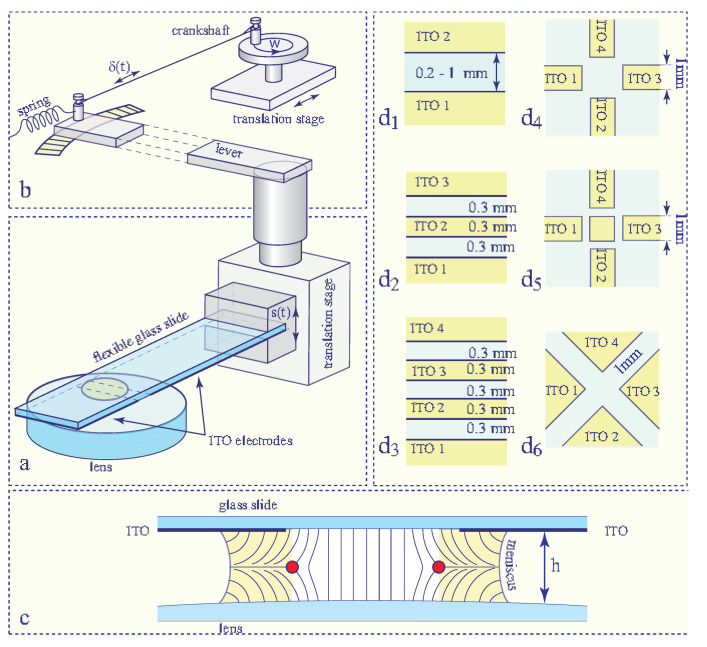
Setup. (**a**) General view. (**b**) Lever-crankshaft system for modulation and control of the gap thickness. (**c**) Cross section through the sample. (**d**) Systems of ITO electrodes.

We will show below that the persistent dowser texture can be generated in nematic droplets od arbitrary sizes by means of a setup tailored especially for this purpose (see Figure 4).

### 6.2. Setup Tailored for Generation of the Dowser Texture

In this setup, the dowser texture is produced in a nematic droplet held by capillarity between a glass slide and a lens with surfaces treated for homeotropic anchoring. The thickness of the drop h is controlled by means of a translation stage equipped with a long lever. After its introduction into the slide/lens gap, the nematic droplet contains always coexisting domains of the dowser and homeotropic textures.

**Figure 5:**
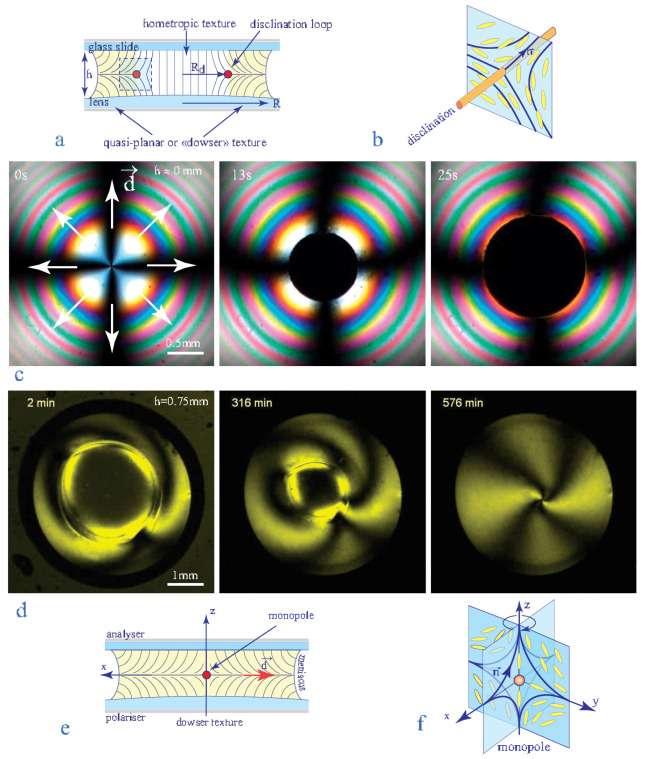
Metastability of the dowser texture. (**a**) Coexistence of the dowser and homeotropic textures. (**b**) Disclination surrounding the homeotropic domain. (**c**) Nucleation and spontaneous expansion of a homeotropic domain inside a thin enough droplet with the dowser texture. (**d**) Spontaneous collapse of the homeotropic domain into a monopole driven by an adequate increase of the sample thickness h. (**e**,**f**) Nematic monopole resulting from the collapse of the disclination loop. **Remark:** Time scales in c and d are very different.

In the simplest case shown in Figure 5c (picture labeled “13 s”) and Figure 5d (picture labeled “2 min”), one homeotropic domain is present in the droplet filled with the dowser texture.

The presence of the coloured interference fringes in pictures of the Figure 5c is due to a very small thickness h (a few micrometers) of the slide/lens gap. In agreement with common observations made during preparation of homeotropic samples [18], the homeotropic domain in such a very thin sample expands at the expense of the dowser texture.

In Figure 5d the nematic droplet contains also one homeotropic domain surrounded by the dowser texture. Here however, the homeotropic domain does not expand but surprisingly shrinks for the benefit of the dowser texture and finally collapses into a point singularity—the nematic monopole. This unusual behaviour confirms observations made by Gilli et al. [4].

The absence of the interference fringes in pictures of Figure 5d is the clue for explanation of such a behaviour: the droplet in Figure 5d is much thicker (h ≈ 2 mm) then the one in Figure 5c.

The thickness h is crucial for the homeotropic ⇒ dowser transition because the total energy of the droplet of radius R containing the homeotropic domain of radius r

(6) $$F_{tot}\approx \frac{K}{2}{\left(\frac{\pi }{h}\right)}^{2}\left(\pi R^{2}-\pi r^{2}\right)h+T2\pi r$$

decreases with the thickness h in its first term representing the distortion energy of the dowser texture while the second term, corresponding to the energy of the disclination of tension T at the dowser/homeotropic interface, is (approximatively) independent of the thickness.

This expression has a maximum at

(7) $$r_{co}\approx \frac{2T}{\pi^{2}K}h\approx 2h$$

In the case of the thin droplet in Figure 5c, the radius r of the homeotropic domain is much larger then r${}_{co}\approx 2h$ so that it expands. On the contrary, in the case of the thick droplet in Figure 5d, the radius r of the homeotropic domain is smaller then $r_{co}\approx 2h$ so that it shrinks and finally collapses.

The recipe for production of the dowser texture is thus very simple: it is enough to increase sufficiently the gap thickness and then wait for the shrinking and the final collapse of the homeotropic domain. In practice, this method is not very convenient because the shrinking-collapse process of a domain with r≈1 mm takes about 10 h as indicated by time labels in Figure 5d.

### 6.3. Flow-Assisted Homeotropic-Dowser Transition, Experiments

To accelerate production of the dowser texture we used currently the method depicted in Figure 6. It consists in submitting a nematic droplet containing one (or more) large homeotropic domain (see Figure 6b-0 s) to a vigorous streaming flow driven by oscillations of the glass slide (see Figure 6b-18 s). This action has two consequences:

1. shredding of the large homeotropic domain into small pieces
2. homeotropic ⇒ dowser transformation induced by the Poiseuille flow

Subsequently, the thickness of the droplet is increased (see pictures labeled 28 s–44 s) so that the radii r of remaining small homeotropic domains are reduced even more. As at the same time the critical radius r${}_{co}$ is increased, the inequality $r<r_{co}$ is reached. After the final collapse of such very small homeotropic domains the thickness of droplet is reduced to its initial value. Let us emphasize that this new production process took 49 s instead of 10 h.

To study the flow-induced homeotropic ⇒ dowser transition in more details, we submitted small homeotropic-in-dowser domains to a slow streaming flow with a well controlled velocity in droplets of 5CB of thickness h = 60 μm. Results are illustrated by the spatio-temporal cross section in Figure 7 which shows how sizes of domains are related to the velocity of the flow. More precisely, in this experiment the velocity of the flow was set to such a value $v_{c}$ that the size r${}_{c}$ of domains was conserved.

Results of such experiments are presented in the plot 1/ r${}_{c}$ versus $v_{c}$ in Figure 7c. From the fit of these data to the linear law

(8) $$1/r_{c}=1/r_{co}\left[1-\left(v_{c}/v_{co}\right)\right]$$

we obtained $r_{co}=69\;\mu$m and $v_{co}=3.9\;\mu$ms${}^{-1}$.

For the sake of clarity let us remind that: (1)—the upper and lower parts of this plot with positive and negative values of the curvature 1/r${}_{c}$ correspond respectively to the complementary cases of the homeotropic-in-dowser and dowser-in-homeotropic domains, (2)—the marginal case of a flat dowser/homeotropic interface with 1/r${}_{c}$ = 0 defines the critical velocity $v_{oc}$, (3)—the critical radius r${}_{co}$ is that of homeotropic-in-dowser domains at rest.

Our experiments on motions and stability of the homeotropic-in-dowser domains were triggered by the recent work of Emeršič et al. [13] who used a microfluidic method to study the flow-induced homeotropic-dowser transition mostly in the complementary geometry of dowser-in-homeotropic domains. Their results, obtained with a channel of thickness h = 12 μm, are also plotted in Figure 7c (with blue crosses) and fitted to the same linear law with $r_{co}=10\;\mu$m and $v_{co}=56\;{\mathrm{\mu ms}}^{-1}$.

**Remark**: The director field lines in the homeotropic texture submitted to hydrodynamic torques in the Poiseuille flow acquires a bow-like shape. The amplitude of this deformation is proportional to the flow velocity. For this reason, the deformed homeotropic texture was dubbed **the bowser texture** by Emeršič et al. [13].

### 6.4. Theory of the Flow-Assisted Homeotropic-Dowser Transition

The explanation of the linear dependence between the critical curvature $1/r_{c}$ of the homeotropic/dowser interface and the flow velocity $v_{c}$ has been proposed first by Emeršič et al. in ref. [13]. More recently, Tang and Selinger developed this theory and presented it in all details in ref. [14]. Here, we will analyse the flow-asssted homeotropic ⇔ dowser transition in slightly different terms.

Let us start with the expression of the hydrodynamic torque per unit volume exerted by a shear flow on the director (see e.g., in ref. [1])

(9) $$\Gamma_{v}=\left[-\alpha_{2}sin^{2}\theta +\alpha_{3}cos^{2}\theta \right]\frac{dv}{dz}$$

We will suppose that the static deformations of the homeotropic and dowser textures due to this torque are so small that they can be neglected. This is true when the Poiseuille flow is slow enough. During expansion of the dowser texture across the strip of width dx (see Figure 7b) the polar angle at the point P decreases from π/2 to πz/h and the torque $\Gamma_{z}$ does the work (per unit volume)

(10) $$w=\int_{\pi /2}^{\pi z/h}\Gamma_{v}d\theta =\frac{dv}{dz}\frac{\left(\alpha_{2}-\alpha_{3}\right)\pi \left(h-2z\right)+\left(\alpha_{2}+a_{3}\right)hsin\left(2\pi z/h\right)}{4h}$$

Knowing that the shear rate in the Poiseuille flow is

(11) $$\frac{dv}{dz}=-\frac{8v_{max}z}{h^{2}}$$

one obtains, after integration on z, the work per unit area

(12) $$W=2\int_{0}^{h/2}w\left(z\right)dz=-v_{max}\left(\frac{\pi (\alpha_{2}-\alpha_{3})}{6}+\frac{\alpha_{2}+\alpha_{3}}{\pi }\right)=v_{max}\left(\frac{\pi \gamma_{1}}{6}-\frac{\gamma_{2}}{\pi }\right)$$

This work corresponds to an effective potential U${}_{eff}$ = −W (see ref. [14]) which has to be added to the total elastic energy given in Equation (6):

(13) $$F^{\prime }=F_{tot}+U_{eff}=\left[\frac{K}{2}\frac{\pi^{2}}{h^{2}}-\frac{v_{max}}{h}\left(\frac{\pi \gamma_{1}}{6}-\frac{\gamma_{2}}{\pi }\right)\right]\left(\pi R^{2}-\pi r^{2}\right)h+T2\pi r$$

The maximum of F’ is located now at

(14) $$\frac{r_{co}}{r_{c}}=1-\frac{v_{max}}{v_{co}}\;\mathrm{with}\;\frac{1}{r_{co}}=\frac{\pi^{2}K}{2Th}\;\mathrm{and}\;v_{co}=\frac{\pi^{2}K}{2h\left(\frac{\pi \gamma_{1}}{6}-\frac{\gamma_{2}}{\pi }\right)}$$

This expression justifies the linear law in Equation (8) used above for the fit of experimental data. **Remark:** Equation (14) and the plot in Figure 7 remain valid for negative values of $v_{max}$ under condition that the dowser field **d** keeps its orientation parallel to the y axis. If an arbitrary direction of the dowser field was imposed f.ex. by an electric field, the linear term in Equation (14) would be $v_{max}\cdot d/v_{co}$.

## 7. Cuneitropism

The dowser texture obtained in droplets of 5CB or MBBA by one of the two methods described above must contain at least one dowson d+. Its presence is required by the homeotropic boundary conditions for the director **n** at the nematic/air meniscus. Indeed, if one examines the structure of the director field in the vicinity of the meniscus shown in Figure 4b it is clear that the dowser field **d** must be orthogonal to the meniscus and have the outward orientation there. On a closed circuit along the circular meniscus, the dowser field rotates by 2π so that the sum of topological charges of all dowsons inside the droplet must be 2π.

In the picture a of Figure 8, only one dowson d+ is present and it is located in the centre of the droplet. The dowser field **d** around it, inferred from the isogyres pattern, has an outward radial configuration. This radial dowser field is orthogonal to concentric isochromes which can be seen as lines of equal thickness h of the lens/slide gap. The radial outward dowser field **d** is thus parallel to the thickness gradient **g** = **grad**h.

In the picture b of Figure 8 the dowson d+ in an eccentric position is connected to the centre of the radial dowser field by a 2π-wall. Experiments show that after about half an hour, the dowson d+ pulled by the shrinking 2π-wall is brought to the droplet centre.

In pictures d and e of Figure 8 the dowson d+ is located in the centre of the droplet and the radial dowser field contains a 2π wall forming a closed loop that shrinks slowly and finally collapses after several hours. The structure of the dowser field inside the 2π-walls is resolved in the pictures c and f.

Both the radial dowser field and the 2π-walls are due to the cuneitropic torque (anticipated in Section 3, see Figure 2c):

(15) $$\vec{\Gamma }=C_{cu}d\times g$$

exerted by the thickness gradient **g**=$\vec{\nabla }h$ on the dowser field **d**.

The origin of this torque can be easily understood with the help of the mechanical model shown in Figure 8a because the distortion of the director field in the dowser texture θ(z) (see Figure 7a) is similar to the deformation θ(z) (see Figure 8g) of the two umbrella ribs inserted in ball bearings which play the role of the homeotropic anchorings.

When the thickness h of the nematic layer is uniform, the director rotates by the angle α=π between the limit surfaces and the elastic energy per unit area is of the order of

(16) $$F_{dow}=\int_{-h/2}^{h/2}\frac{K}{2}{\left(\frac{\alpha }{h}\right)}^{2}dz=\pi^{2}\frac{K}{2h}$$

When the upper surface is tilted by a small angle γ with respect to the lower one, the director rotates between them by the angle $\alpha =\pi -\gamma \cos\psi_{c}$ and one has

(17) $$F_{dow}={\left(\pi -\gamma \cos\psi_{c}\right)}^{2}\frac{K}{2h}\approx \pi^{2}\frac{K}{2h}-\frac{\pi K}{h}\gamma \cos\psi_{c}$$

The cuneitropic torque is thus

(18) $$\Gamma_{cu}=-\frac{dF_{dow}}{d\psi_{c}}=\frac{\pi K}{2h}\gamma \sin\psi_{c}=\frac{\pi K}{2h}d\times g.$$

This equation confirms the anticipated expression of the cuneitropic torque given in Figure 2c with $C=\frac{\pi K}{2h}$.

The thickness h of the gap between the glass slide and the lens’ surface of curvature 1/R${}_{l}$ varies as

(19) $$h\left(r\right)=h_{min}+R_{l}\left[1-\sqrt{1-\frac{r^{2}}{R_{l}^{2}}}\right]$$

so that the thickness gradient **g** is radial, in outward direction, with the amplitude

(20) $$\gamma \left(r\right)=\frac{dh}{dr}=\frac{r}{R_{l}}\frac{1}{\sqrt{1-\frac{r^{2}}{R_{l}^{2}}}}$$

The radial configuration of the dowser field corresponds thus to the equilibrium in which the cuneitropic torque given by Equation (18) vanishes.

This is obviously not the case when 2π-walls are present in the dowser field (see Figure 8b,d). In equilibrium, the non zero cuneitropic torque must be balanced by the elastic torque resulting from the deformation $d^{2}\psi_{c}/d\xi^{2}$ of the dowser field along the axis ξ orthogonal to the wall (see Figure 8c):

(21) $$\frac{Kh}{2}\frac{d^{2}\psi_{c}}{d\xi^{2}}-\frac{\pi K}{2h}\gamma \sin\psi_{c}=0$$

Introducing the characteristic length

(22) $$\xi_{c}=h\sqrt{\frac{1}{\pi \gamma }}$$

this equation can be rewritten as

(23) $$\xi_{c}^{2}\frac{d^{2}\psi_{c}}{d\xi^{2}}-\sin\psi_{c}=0$$

Its solution

(24) $$\psi_{c}\left(\xi \right)=4\arctan\left(e^{-\frac{\xi }{\xi_{c}}}\right)$$

describes the structure of the 2π-wall of width $\xi_{c}$ across which the angle $\psi_{c}$ between the dowser field and the thickness gradient **g** varies by 2π. The energy of the distortion of the dowser field per unit length of the 2π-wall, or equivalently its tension is

(25) $$T=\frac{1}{2}\frac{Kh}{2}\int_{-\infty }^{\infty }{\left(\frac{\partial \psi_{c}}{d\xi }\right)}^{2}d\xi =K\frac{2h}{\xi_{c}}$$

In the case of the eccentric dowson d+ (see Figure 8b), this tension pulls it toward the center of droplet (minimum of the thickness h). In the second case of the circular wall of radius R${}_{w}$ (Figure 8d), the tension T generates the Laplace force T/R${}_{w}$ driving its shrinking.

Let us stress that the cuneitropism of the dowser texture is analogous to the concept of the geometrical anchoring introduced by Lavrentovich [17].

## 8. Electrotropism

### 8.1. Generation of 2π-Walls

The electrotropism anticipated in Section 3 and defined in Figure 2a involves the torque exerted on the polarisation of the dowser field by the electric field:

(26) $$\Gamma_{fe}=P_{fe}d\times E$$

Using the same arguments as above, one expects that the dielectric torque should generate 2π-walls similar to the one in Figure 8b. Confirmation of this expectation was obtained in the experiment represented in Figure 9. Here, a DC electric field generated by the one-gap system of electrodes depicted in Figure 4d1 is applied to a small and thick enough droplet in which the cuneitropic torque proportional to 1/h can be neglected.

Initially (see Figure 9a), the dowson d+ is located in a field-free area. The configuration of the dowser field **d**(x,y) in the whole droplet can be inferred from the isogyres’ pattern. On one hand, it satisfies the boundary condition: **d** orthogonal to the circular edge of the droplet. On the other hand, the dowser field inside the gap between electrodes is parallel to the electric field: **d**//**E**. This means that the polarisation aligned by the electric field is parallel to the dowser field: $P_{fe}=P_{fe}d$ with $P_{fe}>0$.

Upon the reversal of the electric field (see Figure 9b), two π-walls, generated at the lateral edges of the gap and converging to the centre, mediate the reversal of the dowser field inside the gap. Upon their junction in Figure 9c they form the expected 2π-wall. Due to its tension T this wall pulls on the dowson d+ and sets it in motion in the direction of the field. The dowson stops to move after the complete contraction of the 2π-wall i.e., when it reaches a new equilibrium position close to the other edge of the gap (see Figure 9e). Upon the second reversal of the electric field an analogous sequence of events is visible in Figure 9f–i.

The width of the wall generated by the electric field results from the balance of the elastic and dielectric torques

(27) $$\frac{Kh}{2}\frac{d^{2}\psi_{fe}}{d\xi^{2}}-P_{fe}E\sin\psi_{fe}=0$$

Using the same calculations as in the previous section one obtains:

(28) $$\xi_{fe}=\sqrt{\frac{Kh}{2P_{fe}E}}$$

Knowing K, h and E, the value of the polarisation P${}_{fe}$ can be determined from measures of $\xi_{fe}$.

### 8.2. Flexo-Electric Polarisation of the Dowser Texture

The search for the electrotropism of the dowser texture was inspired by the pioneer work of R.B. Meyer who in 1969 remarked that the $D_{\infty h}$ symmetry of nematics is broken when the director field **n** is distorted [19]. Using symmetry arguments, Meyer postulated that a polarisation density **p**${}_{fe}$ expressed (using notations of ref. [1]) as

(29) $$p_{fe}=e_{1}n\vec{\nabla }\cdot n+e_{3}\left(\vec{\nabla }\times n\right)\times n$$

is allowed in a distorted nematic. As this polarisation is not spontaneous but induced by the distortion of the director field, it was subsequently called **flexo-electric**.

In the case of the dowser texture with the director field given in Equation (3), one obtains, after integration on z, the polarisation per unit area

(30) $$P_{fe}=\int_{-h/2}^{h/2}p_{fe}dz=P_{fe}d$$

with

(31) $$P_{fe}=\frac{\pi }{2}\left(e_{3}-e_{1}\right)$$

The flexo-electric polarisation of the dowser texture is thus is independent of the thickness h.

Let us note that the expression (30) can be obtained without calculation through following symmetry considerations. The global symmetry of the dowser texture $C_{2v}$, lower than that of a undistorted nematic $D_{\infty h}$, contains only three elements: the axis $C_{\infty }$ and two orthogonal mirror planes passing through it. The mirror plane σ passing through the z axis is indicated in Figure 1c while the second mirror plane orthogonal to the z axis is not indicated for the sake of clarity. Such a low symmetry allows the dowser texture to posses a polarisation which for symmetry reasons must be collinear with the $C_{\infty }$ axis i.e., with the dowser field **d**.

### 8.3. Motions of Dowsons in an Homogeneous Electric Field

In the experiment discussed above, the motion of the dowson d+ was interpreted as driven by the tension T of the 2π wall generated by the DC electric field. In these considerations we assumed implicitly that the wall reached already its quasi-static configuration. One can ask nevertheless what would be the motion of the dowson d+ in an alternating electric field with a period much shorter than the characteristic time required for formation of walls. Moreover, the dowser field surrounding the dowson d+ can adopt a continuous set of configurations (a few examples of them are given in Figure 10b–g) different from the radial outward one discussed previously. One can ask therefore whether the motion of the dowson d+ in the electric field is related to its topological charge +2π or, on the contrary, depends on its configuration. The same issue concerns dowsons d− (see Figure 10h–j).

All possible configurations of dowsons can be expressed as

(32) d=(cosφ,sinφ) with φ=mψ+δ

with ψ—the angle of polar coordinates (see Figure 10a), φ—the angle between the dowser feld **d** and the x axis, δ—an angular variable and m = +1 or −1.

When an electric field **E** parallel to the y axis is applied to a dowson, its interaction with the polarisation **P** is expressed by the density of energy per unit area

(33) $$F=-P_{fe}d\cdot E$$

In the sector of the angular width dψ and length r and making the angle ψ with the x axis (see Figure 10k), the dielectric energy is thus

(34) $$\delta F=-P_{fe}d\cdot E\frac{r^{2}}{2}d\psi$$

so that there is a force

(35) $$\delta f=\frac{d(\delta F)}{dr}\left(\cos\psi ,\sin\psi \right)=-P_{fe}d\cdot Er\left(\cos\psi ,\sin\psi \right)d\psi$$

acting on the dowson. Using the expression (32) of the dowser field around dowsons one obtains the total force by integration on ψ:

(36) $$f=-\int_{0}^{2\pi }P_{fe}E\sin\left(m\psi +\delta \right)\left(\cos\psi ,\sin\psi \right)d\psi =P_{fe}E\pi r\left(\sin\delta ,-m\cos\delta \right)$$

Directions of the force **f** indicated in Figure 10 correspond to the case of $P_{fe}>0$. In particular the force **f** acting on the dowson d+ in its radial outward configuration in Figure 10b is antiparallel to the electric field like in the experiment with 5CB depicted in Figure 9.

The dowser field around the dowson d+ can reach the radial outward configuration, satisfying both the boundary condition and the action of cuneitropism, through the spontaneous viscoelastic relaxation. This process which can last for a few hours in a droplet with the radius in the range of a few mm, can be made almost instantaneous by application of a diverging Poiseuille flow accompanying reduction of the slide/lens gap thickness (see the next section on rheotropism). By the same rheotropic mechanism, the converging Poiseuille flow driven by an increase of the gap thickness can force the radial inward configuration. As this inward configuration is unstable it must return by viscoelastic relaxation to the outward one. Nevertheless, as the characteristic time of the relaxation process is of the order of several hours, experiments testing the validity of Equation (36) can easily be made.

Results of experiments with the diverging and converging configurations of the dowson d+ in MBBA are summarised in Figure 11. The spatio-temporal cross sections in the last column show that directions of motions are reversed between the two radial configurations. As they are also reversed with respect to those in Figure 10b,c we can conclude that the flexo-electric polarisation in MBBA $P_{fe}$ is antiparallel to the dowser field **d**. The same experiments performed with 5CB agree directly with theoretical schemes in Figure 10b,c which means that in 5CB $P_{fe}$ is parallel to the dowser field **d**.

The zig-zag shaped trajectories of the dowson d+ in spatio-temporal cross section in Figure 11 show also that the direction of motion is reversed instantaneously upon a reversal of the electric field. Assuming that the velocity of motion is proportional to the driving force given in Equation (36), this feature also agrees with theoretical predictions.

### 8.4. Effects Due Electro-Osmotic Flows

Beside trajectories of dowsons d+ the spatio-temporal cross sections in Figure 11 display also zig-zag shaped trajectories of dust particles transported by electro-osmotic flows which in MBBA are parallel to the electric field. The same experiments performed with 5CB confirmed the presence of electro-osmotic flows but in the direction antiparallel to the electric field.

Let

(37) $$\vec{Q}=-\int_{-h/2}^{h/2}{\vec{v}}_{eo}\left(z\right)dz$$

be the global flux of the electro-osmotic flow driven by the electric field inside the gap. The velocity profile ${\vec{v}}_{eo}\left(z\right)$ can be quite complex and we have no place to discuss it here. Due to incompressibility of nematics, the flux $\vec{Q}$ must be conserved in field-free areas adjacent to the gap where the velocity profile, in the absence of body forces, must be that of a Poiseuille flow. Experiment with 5CB illustrated by the series of three pictures the Figure 12 shows indeed that this secondary Poiseuille flow generates 2π-walls connected to dowsons similar to those generated by the cuneitropic and electrotropic torques.

## 9. Rheotropism

### 9.1. Hydrodynamic Torque

Generation of 2π-walls by homogeneous flows as well as of the radial dowser fields by diverging or converging flows unveil the alignment of the dowser field **d** by Poiseuille flows. This phenomenon, similar to the action of winds on a weather vane and called rheotropism is due to the hydrodynamic torque $\Gamma_{z}$ exerted by velocity gradients dv/dz on the director field **n** given in Equation (3).

With $\psi_{r}$—the angle between the dowser field **d** and **v**(z)- velocity of the Poiseuille flow (see Figure 2b) the expression of the Leslie-Ericksen hydrodynamic torque (see ref. [1]) per unit area is:

(38) $$\Gamma_{z}=-\int_{-h/2}^{h/2}\alpha_{2}\frac{\partial v}{\partial z}\sin\psi_{r}\sin\theta \left(z\right)\cos\theta \left(z\right)dz$$

Knowing that in the Poiseuille flow the velocity profile is given by

(39) $$v=v_{max}\left(1-\frac{z^{2}}{{\left(h/2\right)}^{2}}\right)\;\mathrm{with}\;\theta =\frac{\pi z}{h}$$

one obtains the expression of the rheotropic torque [9]

(40) $$\Gamma_{rh}=-\frac{2\alpha_{2}}{\pi }v_{max}\sin\psi_{r}=-\frac{2\alpha_{2}}{\pi }d\times v_{max}$$

similar to those of the cuneitropic and electrotropic torques in Equations (18) and (26). From the equation of the balance of $\Gamma_{rh}$ with the elastic torque

(41) $$\frac{Kh}{2}\frac{d^{2}\psi_{c}}{d\xi^{2}}-\frac{2|\alpha_{2}|}{\pi }\sin\psi_{c}=0$$

one obtains the width of the 2π-wall

(42) $$\xi_{rh}=\sqrt{\frac{\pi Kh}{4|\alpha_{2}|v_{max}}}$$

which decreases as $\sqrt{1/v_{max}}$.

### 9.2. Winding of the Dowser Field

In the experimental setup show in Figure 4, changes in the lens/slide gap’s thickness h due to vertical translations s(t) of the slide’s support are accompanied by flexion of the slide submitted to viscous stresses $\sigma_{zz}$ inside the nematic droplet. The viscous stresses are proportional to the velocity dh/dt of the thickness variations. Therefore, in the case of a harmonic excitation $s\left(t\right)=s_{o}\cos\left(\omega t\right)$, the simultaneous flexural and translational motions of the glass slide are shifted in phase by π/2.

With the sake of simplicity, we show in Figure 13a an idealised sequence composed of pure translations and pure tilts of the glass slide. Translations drive radial inward and outward flows while tilts drive dipolar flows. Let us consider flows occurring during the following sequence of four motions of the glass slide: S = compression ⇒ tilt+ ⇒ dilation ⇒ tilt-. Obviously, at the end of this sequence the initial configuration of the slide/lens pair is restored. However, if one considers the sequence of flows—radial outward ⇒ dipolar ⇒ radial inward ⇒ dipolar inverted—it may result in rotation by 2π of the dowser field when the amplitude of flows is large enough. The amplitudes of the radial and dipolar flows varies with the position (x,y) inside the droplet. In the two areas indicated by dashed squares these amplitudes are large so that the dowser field rotates by 2π there. As this rotation is respectively clockwise and anticlockwise in the right and left halves of the droplet, one sequence S must generate two 2π-walls.

Result of an experiment performed with the harmonic excitation s(t) is illustrated by the series of four pictures in Figure 13b. The first two pictures show that two walls generated by the first sequence S form donut-shaped closed loops. Two next sequences generate new pairs of similar 2π-walls.

The winding of the dowser texture has been realised also with other setups tailored especially for this purpose. Their descriptions can be found in references [9] and [11].

## 10. Generation of Dowsons

Unlike disclinations which are ubiquitous and easy to generate, monopoles—topological point defects of the director field—were considered as scarce and difficult to generate in bulk nematics for topological reasons. Indeed, the director field **n** on a sphere surrounding a monopole mapped on the space of the nematic order parameter covers it twice (see Figure 14e). Such a configuration of the director field is unlike to occur spontaneously. Controlled generation of monopoles was reported for the first time by Nikkhou et al. [20] by means of a local Isotropic ⇒ Nematic quench in vicinity of a glass fibre.

In the dowser texture, nematic monopoles are much easier to generate because their 3D director field **n**(z) has already a built-in permanent distortion—rotation of **n** by π between limit surfaces. Let us map the director field of the dowser texture represented in Figure 14e-0${}^{\circ }$ on the sphere equivalent to a sum of two spaces of the nematic order parameter. Figure 14e shows that this mapping covers the Prime meridian. To meet topological conditions favorable for generation of monopoles the dowser texture must be wound up. Indeed, when a 2π-wall of the wound up dowser texture is crossed (see Figure 14d) the space of the nematic order parameter is effectively covered twice.

In these conditions, generation of monopoles pairs, corresponding to pairs of dowsons (d+,d−), occurs when 2π-walls are broken.

Experiments have shown that 2π-walls are breaking spontaneously when they are thin enough because their tension is inversely proportional to their width ξ (see Equation (25)). We know also from Section 9.1 and Section 8.1 that the walls’ width ξ depends on the velocity v${}_{o}$ of the Poiseuille flow (Equation (42)) or on the intensity E of the electric field (see Equation (28)).

For this reason, when the wound up dowser texture shown in Figure 14a is submitted to a strong electric field, applied by means of the one-gap system of ITO electrodes (see Figure 14b), the 2π-walls become so thin that they break and two rows of dowsons d+ and d− are created (see Figure 14c).

**Figure 6:**
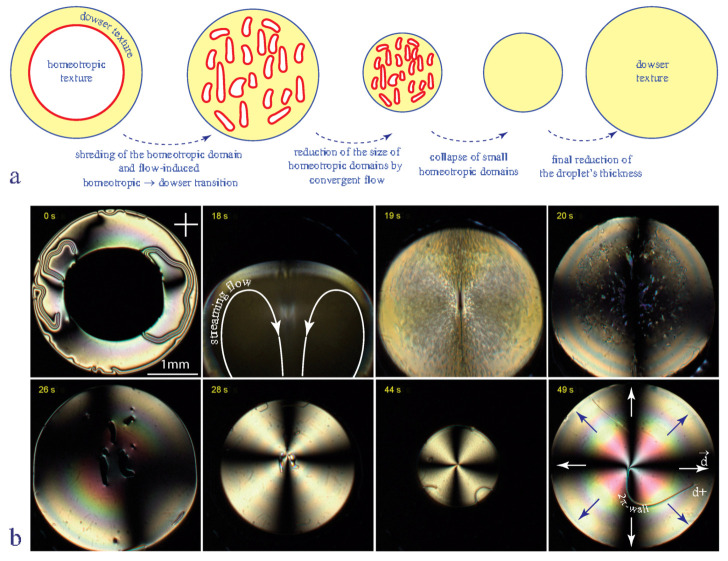
Homeotropic ⇒ Dowser transformation accelerated by a streaming flow driven by oscillations of the glass slide. (**a**) Schematic representation of four stages of the method used for a rapid preparation of the dowser texture. (**b**) Series of eight pictures taken during the process (5CB).

**Figure 7:**
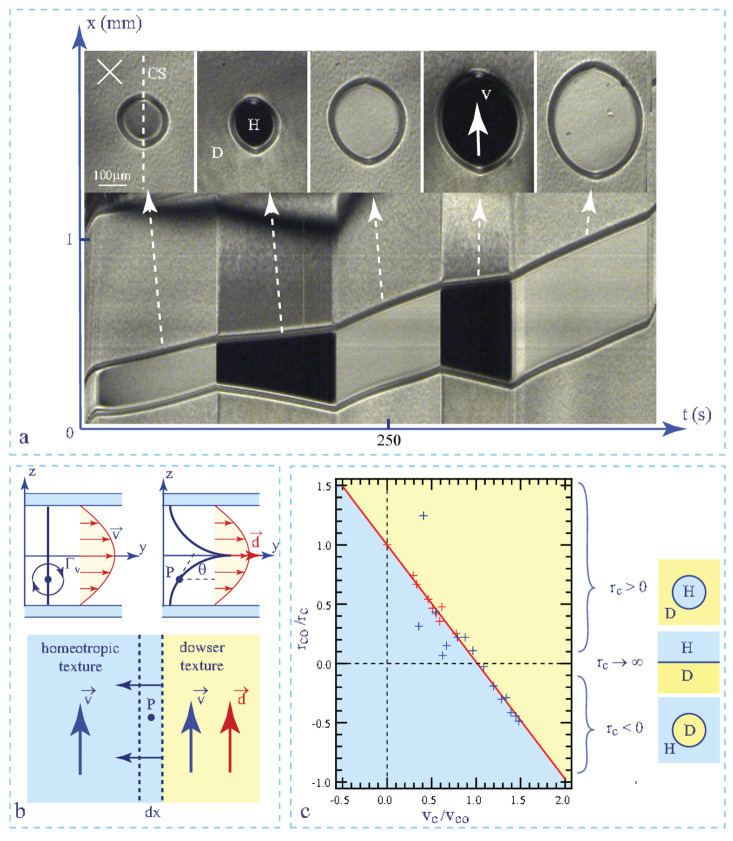
Stability of homeotropic-in-dowser domains in Poiseuille flows. (**a**) Spatio-temporal cross section showing the motion of a homeotropic-in-dowser domain. (**b**) Homeotropic and dowser textures submitted to a Poiseuille flow. (**c**) Stability diagram. Red crosses—experimental points extracted from the spatio-temporal cross sections such as the one in a. Blue crosses—experimental data from Figure 2G in ref. [13]. Red plain line—fit to the Equation (8). (5CB, h = 60 μm).

**Figure 8:**
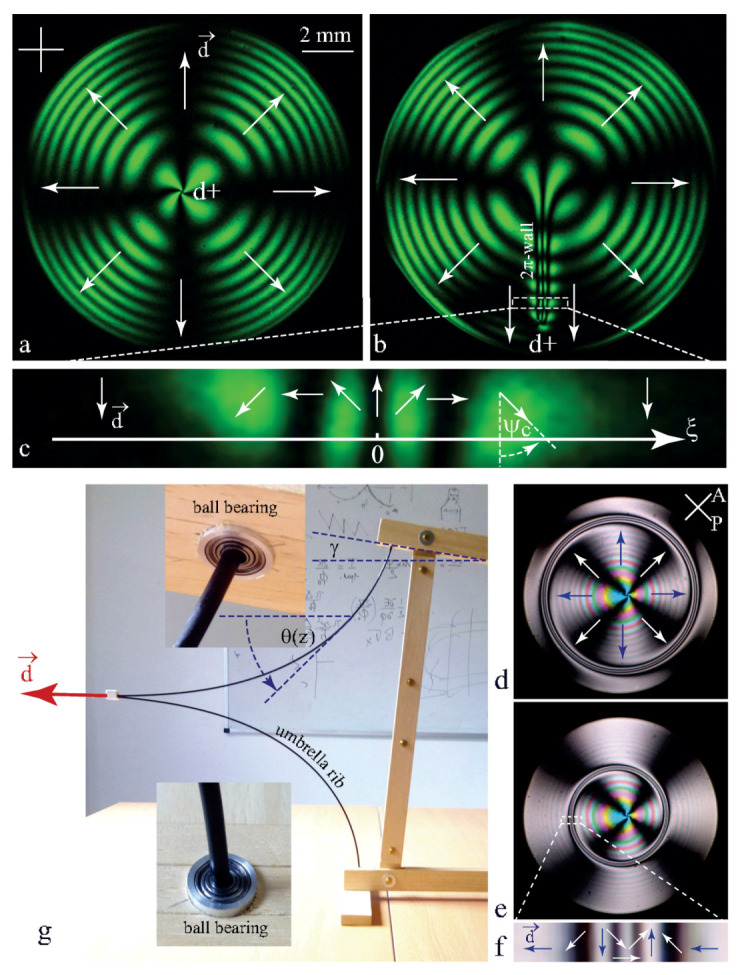
Cuneitropism. (**a**) Radial dowser field with **d**//**grad**h. (**b**) 2π-wall connecting the eccentric dowson d+ with the center of the radial dowser field. (**d**,**e**) Radial dowser field with a shrinking circular 2π-wall. (**c**,**f**) Structures of 2π-walls. (**g**) Mechanical model explaining the cuneitropism of the dowser texture.

**Figure 9:**
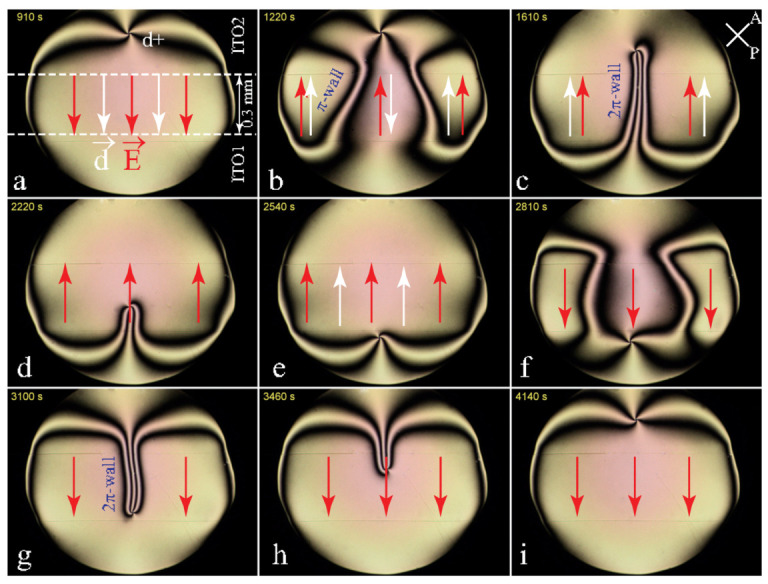
The evidence for the polarisation of the dowser texture in 5CB: formation of a 2π-wall upon application of the electric field to the sample containing one dowson d+. (**a**) Static texture in the presence of the electric field. (**b**) Formation of π-walls after reversal of the electric field. (**c**) Formation of a 2π-wall from two π-walls. (**d**) Motion of the dowson d+ pulled by the 2π-wall. (**e**) New static texture of the dowser field. (**f**–**i**) Evolution of the dowser field after the second reversal of the electric field.

**Figure 10:**
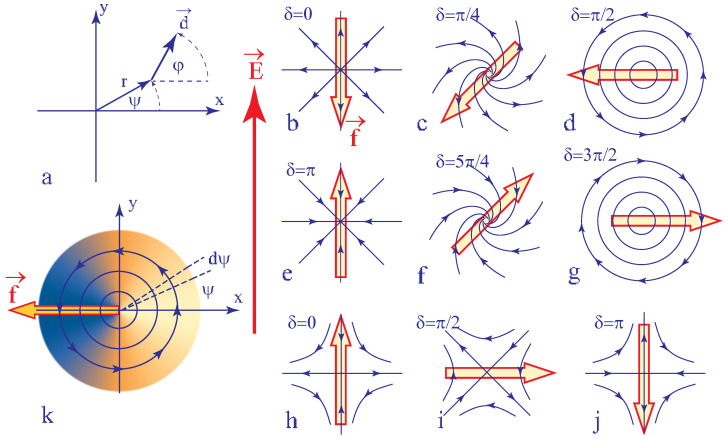
Configurations of dowsons d+ and d− and their motions in electric field. Direction of forces **f** indicated here are given by Equation (36) with $P_{fe}>0$. (**a**) Definition of variables r, ψ and φ. (**b**–**j**) Direction of the force action on dowsons in several configurations. (**k**) Energy density (given in Equation (33)) in the orthoradial configuration of the dowson d+.

**Figure 11:**
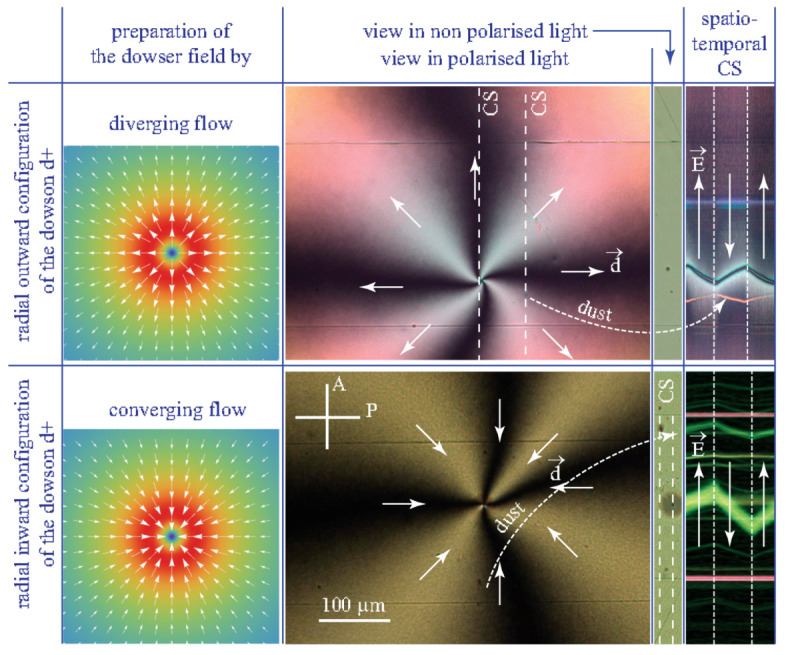
Electric field-induced motion of a dowsons d+. The radial outward and radial inward configurations of the dowson are generated by diverging and converging Poiseuille flows. For the same direction of the electric field, the direction of motion of dowson is opposite for the outward and inward configurations. The motion of the dust particles is due to electro-osmotic flows. (MBBA).

**Figure 12:**
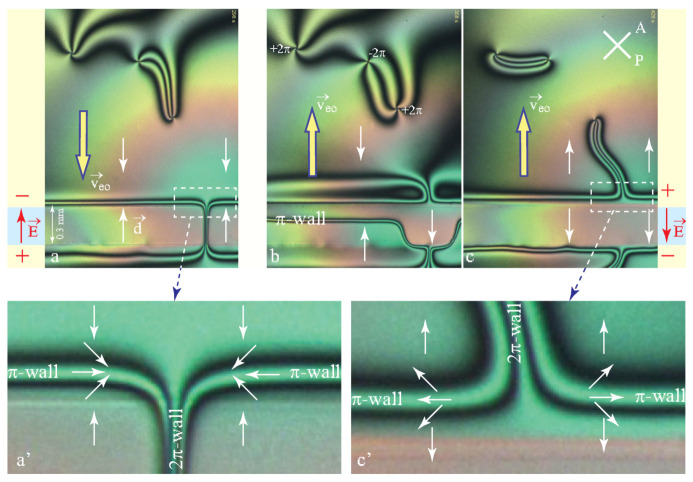
Simultaneous evidence for the flexo-electric polarisation of the dowser texture and for the electro-osmotic flows in 5CB. (**a**,**a’**) Application of an electric field by the one-gap system of electrodes. Due to its flexo-electric polarisation, the dowser field **d** is aligned by the electric field **E** inside the gap. π-walls are generated at edges of the gap because outside of the gap, the dowser field **d** is aligned in the opposite direction by the electro-osmotic flow **v**${}_{eo}$. (**b**) Transitory splitting of the 2π-wall and motions of π-walls due the reversal of the electric field. (**c**,**c’**) Alignment of the dowser field by the reversed electric field and electro-osmotic flow.

**Figure 13:**
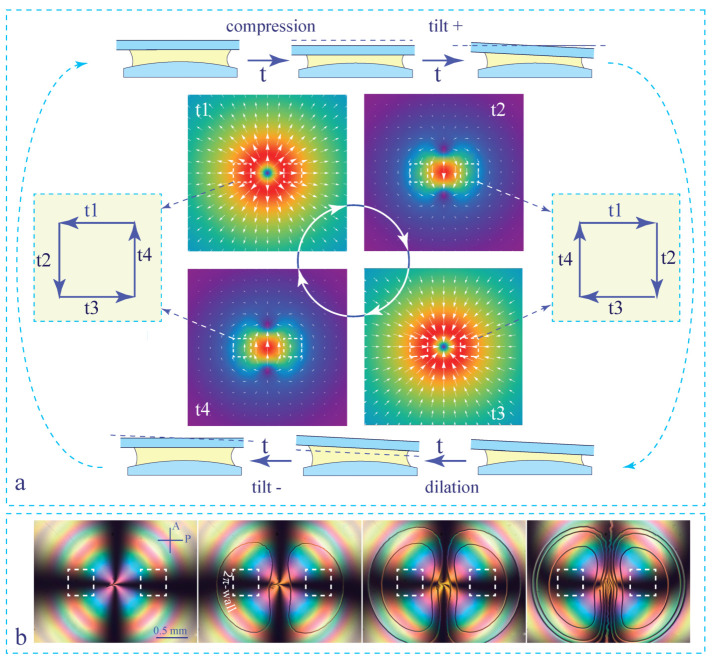
Winding of the dowser field by a sequence of radial and dipolar flows. (**a**) One sequence of radial and dipolar flows appropriate for the winding process. (**b**) Generation of three 2π-walls loops by three successive sequences of flows.

**Figure 14:**
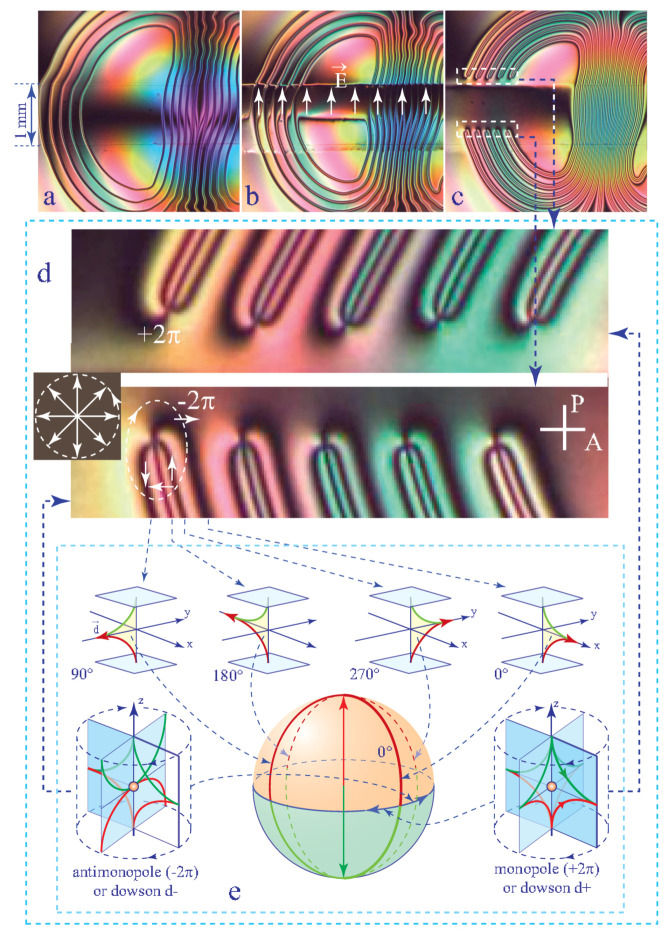
Generation of dowsons equivalent to nematic monopoles. (**a**) Wound up dowser texture made of 2π-walls. (**b**) Thinning of the 2π-walls in a strong electric fields leads to their breaking. (**c**) Two rows of dowsons d+ and d− generated by breaking of the 2π-walls. (**d**) Detailed views of the dowser field. (**e**) Mapping of the director field on the space of the nematic order parameter.

Thinning of the 2π-walls leading to their breaking can also be obtained by application of strong flows to wound up dowser textures [11]. In the extreme case of a turbulent streaming (similar to the one used to eliminate the homeotropic texture in Figure 6) applied to a tightly wound up dowser texture, one obtains a complex pattern of dowsons d+ and d− which will be discussed in the next section. Their total topological charge imposed by the orthogonal boundary conditions at the nematic/air meniscus is +2π.

## 11. Stabilisation of Dowsons Systems by Inhomogeneous Electric Fields

If this complex configuration of dowsons was allowed to relax in the presence of the cuneitropic torque due to the plane/sphere geometry of the gap, annihilations of dowsons pairs (d+,d−) would lead the dowser field to its ground state - the radial outward configuration with one dowson d+ in the centre.

**Figure 15:**
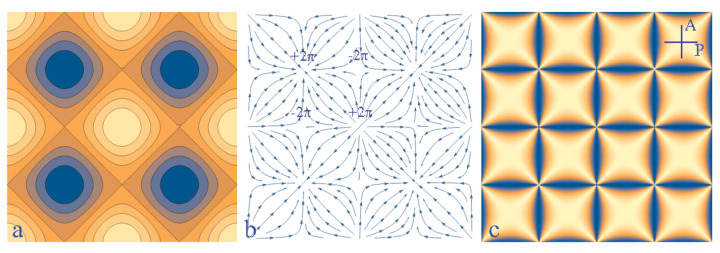
Gadanken experiment: stabilisation of a dowsons pattern by tropisms of the dowser texture. (**a**) Topography of the gap thickness h(x,y) or of the electric potential U(x,y). (**b**) Dowser field **d** oriented by the corresponding thickness gradient g=∇h or electric field E=∇U. (**c**) Pattern of isogyres seen in polarising microscope.

However, if the gap thickness h(x,y) had a more complex topography such as the one represented in Figure 15a, the cuneitropic torques could align the dowser field **d** along the thickness gradient **g** shown in Figure 15b and a square network of alternating dowsons d+ and d− could be stabilised by this means.

Similarly, the same network of dowsons could be stabilised by the electric field **E** (Figure 15b) derived from the periodic potential U(x,y) represented in Figure 15a.

In such a periodic potential, stable positions of dowsons d+ and d− are located respectively in extrema and in saddle points.

### 11.1. Triplet of Dowsons Stabilised in MBBA by a Quadrupolar Electric Field

**Figure 16:**
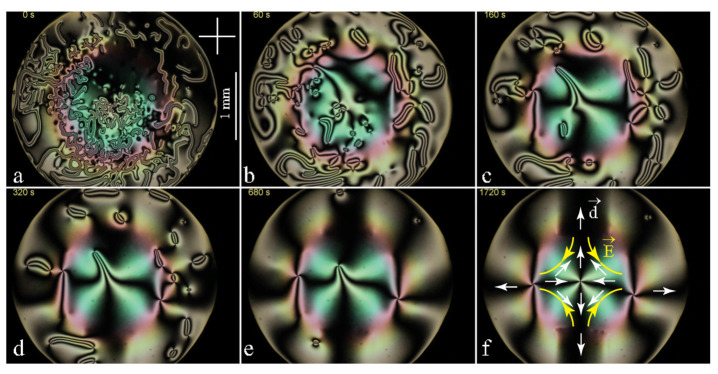
Triplet of dowsons stabilised by a quadrupolar electric field. (**a**) Initial configuration. (**a**–**f**) Relaxation through annihilation of dowsons pairs. (**f**) Final triplet configuration stabilised by the electric field. In MBBA, the dowser field is antiparallel to the electric field.

As such a periodic electric field is difficult to realise by means of etched ITO electrodes, a much simpler system of four electrodes from Figure 4d4 has been used. The electric field generated by it is analogous to the flow field in a cross junction of four microfluidic channels used by Giomi et al. [12]. The series of six pictures in Figure 16 shows that the viscoelastic relaxation occurring in the presence of the quadrupolar electric field leads to the stable triplet of dowsons shown in Figure 16f.

As expected, the dowson d− is located in the stagnation point of the electric field (saddle point of the potential U). From the isogyres’ pattern Figure 16f it is possible to infer that the dowser field **d** around this dowson d− is antiparallel to the electric field **E**. It is so because, as we know from Section 8, in MBBA the flexo-electric polarisation $P=P_{fe}d$ is antiparallel to **d**.

### 11.2. Septet of Dowsons in MBBA Stabilised by a Quadrupolar Electric Field

**Figure 17:**
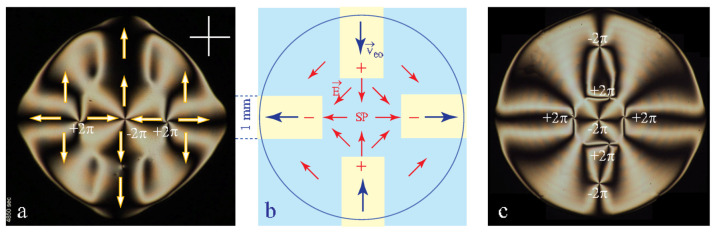
Triplet and septet of dowsons. The septet configuration is stabilised by electro-osmotic flows which in MBBA are parallel to the electric field. (**a**) Triplet of dowsons. (**b**) Geometry of the electric field. (**c**) Septet of dowsons.

Figure 17 shows that in a stronger electric field, beside the triplet state (Figure 17a) also a septet of dowsons can be stabilised by the quadrupolar electric field (Figure 17b). It contains the same triplet of dowsons as in Figure 17a and two additional pairs of dowsons which are located in field-free areas above the two positive ITO electrodes.

In the absence of the electric field, annihilation of these two dowsons pair is hindered by electro-osmotic flows indicated in Figure 17b. In MBBA they have the same direction as the electric field in the adjacent gap.

### 11.3. Dowsons d+ Stabilised by Corner Singularities of the Electric Field

**Figure 18:**
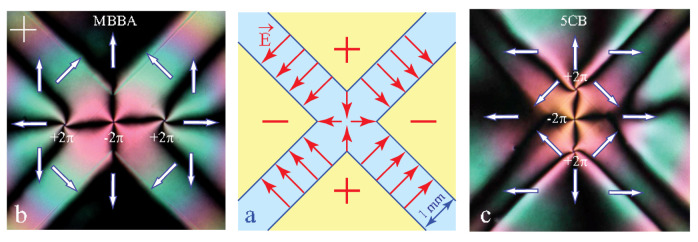
Stable configurations of a dowsons’ triplet in a quadrupolar electric field. (**a**) Electric field generated by the system of four ITO electrodes. (**b**) The dowsons’ triplet in MBBA. The dowson d− is trapped in stagnation point of the electric field. (**c**) The same experiment with 5CB.

Quadrupolar electric field with the stagnation point stabilising the dowson d− can be generated also with the system of electrodes d${}_{6}$ shown in Figure 18a (see also Figure 4d6). It displays a pertinent difference with the system d${}_{4}$: beside the central stagnation point it contains also four point singularities of the electric field at corners of electrodes which are susceptible to stabilise dowsons d+.

As expected, in an experiment realised with MBBA (see Figure 18b) the two dowsons d+ of the triplet configuration are located at sharp corners of negatively charged electrodes. In the same experiment realised with 5CB, the triplet of dowsons has a different orientation because the flexo-electric polarisation **P**${}_{fe}$ in 5CB is parallel do the dowser field **d** while in MBBA it is antiparallel to **d**.

## 12. Conclusions and Perspectives

Thanks to new methods tailored for generation and handling of the dowser texture the knowledge of its properties is progressing and new phenomena are unveiled. In this paper we focussed mainly on the first order sensitivity of the dowser texture to fields acting on it. We have also shortly outlined several other topics such as generation of dowsons, their sensitivity to fields and stabilisation of dowsons systems by inhomogeneous fields. This last issue deserves certainly more detailed studies. In particular, the ”gedanken” experiment proposed in Section 11 (see Figure 15) should be tempted.

We have also shortly discussed the sensitivity of the homeotropic⇔dowser transition to Poiseuille flows. A more detailed study of this issue is under way by means of the circular dowsons collider CDC2 in which an orthoradial streaming flow is parallel to a circular homeotropic/dowson interface. Let us remind that the homeotropic⇔dowser transition is of prime importance for microfluidic studies [12,13] involving an interplay between “bowsons” i.e., defects of the bowser texture (homeotropic texture deformed by Poiseuille flows) and dowsons, defects of the dowser texture.

**Figure 19:**
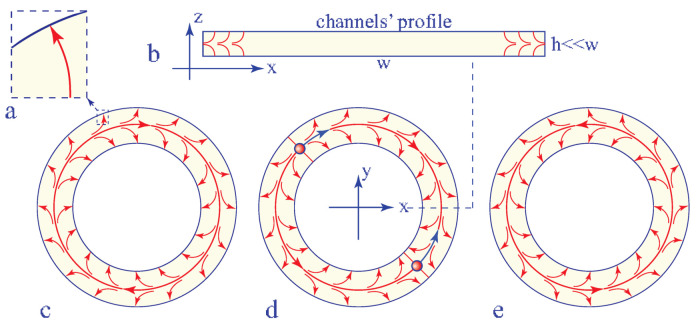
Dowser field in an annular channel. (**a**) Cross section of the channel. (**b**) Homeotropic outward anchoring of the dowser field at the lateral wall of the channel. (**c**,**e**) Defect-less dowser fields with opposite curls. (**d**) Dowser field in the presence of a dowsons’ pair d+d−.

Studies of the dowser field in non simply-connected topologies, i.e., other that the trivial one of a disc, remain to be done. This new direction appears as promising because in a single annular channel or in more complex networks of channels connected by n-junctions, the structure and topology of the dowser field **d** are imposed by anchoring conditions at lateral walls of channels. When the anchoring of the director field at lateral walls of channels is the homeotropic one (see Figure 19a), the resulting preferential orientation of the dowser field **d** can be qualified as a “**homeotropic outward**” i.e., orthogonal to lateral walls and oriented in outward direction (see Figure 19b). In the case of an annular channel, the total topological charge of the dowser field imposed by these boundary conditions is zero. In the absence of dowsons pairs, the dowser field **d** in such a single annular channel is non trivial because it must have in average either a clockwise or an anticlockwise curl (see Figure 19c,e). The sign of the curl can be reversed by a three-stage process: 1—generation of a dowsons pair d+d−, 2—circular motion of dowson in opposite clockwise and anticlockwise direction (see Figure 19d), 3—annihilation of dowser pair.

We hope that thanks to the analogy with other systems (f.ex. superconductors) that share with the dowser texture the same order parameter, the studies of the dowser texture are of interest not only to experts in liquid crystals.
